# Preoperative evaluation of the efficacy of radio-hyperthermo-chemotherapy for soft tissue sarcoma in a case series

**DOI:** 10.1371/journal.pone.0195289

**Published:** 2018-04-16

**Authors:** Hisaki Aiba, Satoshi Yamada, Jun Mizutani, Norio Yamamoto, Hideki Okamoto, Katsuhiro Hayashi, Hiroaki Kimura, Akihiko Takeuchi, Shinji Miwa, Takashi Higuchi, Kensaku Abe, Yuta Taniguchi, Yoshihiro Araki, Hiroyuki Tsuchiya, Takanobu Otsuka

**Affiliations:** 1 Department of Orthopaedic Surgery, Nagoya City University Graduate School of Medical Sciences, Nagoya, Aichi, Japan; 2 Department of Orthopaedic Surgery, Graduate School of Medical Sciences, Kanazawa University, Kanazawa, Ishikawa, Japan; University of Texas M. D. Anderson Cancer Center, UNITED STATES

## Abstract

**Purpose:**

Radio-hyperthermo-chemo (RHC) therapy, which combines radiotherapy, hyperthermia, and chemotherapy, for malignant soft tissue tumors has been introduced with the aim of decreasing the possibility of local recurrence after surgery. To avoid unnecessary neoadjuvant therapy and to plan the appropriate surgical treatment, surveillance of RHC therapeutic efficacy during treatment is necessary. In this study, we determined the optimal response criteria to evaluate the efficacy of RHC by comparing preoperative images before and after RHC with pathological evaluation of necrosis in the resected tumor.

**Patients and methods:**

From 2004 to 2014, 20 patients were enrolled into this study. Needle biopsy revealed 6 cases of myxoid liposarcoma, 6 cases of undifferentiated pleomorphic sarcoma, 4 cases of myxofibrosarcoma, and 4 cases of synovial sarcoma. Based on the Response Evaluation Criteria in Solid Tumors (RECIST) 1.1 or modified RECIST, we calculated the responses to RHC therapy by comparing pre- and post-RHC therapy images. In addition, resected specimens underwent pathological analysis to evaluate response based on tumor necrosis. The correlation between assessment based on preoperative images and resected tumors were evaluated by the Spearman’s rank-order correlation coefficient.

**Result:**

From the surgical specimens, pathological assessment of necrosis in resected tumor were assessed as less than 50% (2 cases), 50–90% (9 cases), 90–99% (6 cases), and total necrosis (3 cases). Use of the RECIST 1.1 underestimated good responders as stable disease (SD) or progressive disease (PD) in 5 out of 15 cases; on the other hand, use of the modified RECIST did not underestimate the pathological assessment of necrosis. The correlations between responses based on preoperative images and those based on histological assessments were 0.23 (RECIST 1.1) and 0.76 (modified RECIST).

**Conclusion:**

Because pathological responses can be underestimated using the RECIST 1.1, the modified RECIST, which take into consideration tumor viability, as assessed by contrast MRI, should also be considered when evaluating the efficacy of RHC.

## Introduction

Soft tissue sarcoma (STS) is an extremely rare disease, with an annual incidence of 3 per 100,000 people, accounting for ≤1% of all malignant tumors [1]. Surgical resection is required to achieve good local control of STS. Although the data from the National Cancer Institute’s Surveillance, Epidemiology, and End Results (SEER) database indicate that 5-year overall survival rates have increased from 28% in 1991–1996 to 62% in 2004–2010 [2], STS remains as one of the most difficult diseases to treat.

To improve the rate of tumor progression beyond the surgical site, we have performed a multidisciplinary approach for local control termed radio-hyperthermo-chemo (RHC) therapy, which combines radiotherapy, hyperthermia, and chemotherapy, at Nagoya City University Hospital beginning in the early 1990s [3]. Although conflicting views exist concerning the roles of neoadjuvant or adjuvant therapy with chemotherapy, radiotherapy, or hyperthermia in the treatment of STS [4,5,6], we have administered this therapy based on evidence from basic and clinical research. When combined with chemotherapy, hyperthermia is thought to enhance the anti-tumor activity of agents such as bleomycin [7], cisplatin [8], and Adriamycin [9, 10] by inhibiting their excretion and/or augmenting cancer cell sensitivity to these agents. In addition, both in vitro and in vivo studies have shown that hyperthermia has synergistic effects with radiotherapy. Hyperthermia compensates for the gaps in the cytotoxicity of radiotherapy by killing cells in S-phase; these cells are generally resistant to radiotherapy, but susceptible to hyperthermia [11]. Moreover, hypoxic cells, which are also impervious to radiotherapy, are vulnerable to hyperthermia because anaerobic metabolism creates a low-pH environment around hypoxic cells, which enhances thermal effects [12]. In the clinical setting, Issels et al. performed a multicenter randomized phase 3 trial in STS and revealed a significant improvement in patient outcomes associated with hyperthermia compared to conventional chemotherapy [13].

We previously reported favorable results with RHC therapy [3]. From 1990–1999, we treated 44 patients with STS of the limbs. Tumor shrinkage was observed in 98% (43/44) of patients, and local recurrence occurred in only 1 patient despite 5 cases undergoing intralesional excision. Following this publication, we have kept performing RHC and reported better local control effect compared to standard therapy. Also, the reduction of surgical margin was achieved by shrinking tumors preoperatively and an amputation was averted in some cases [14, 15].

Surveillance of RHC therapy efficacy during treatment is necessary, as administration of nonbeneficial neoadjuvant therapy and consequential postponement of surgery, can be harmful to patients. Preoperatively, imaging can provide valuable information for monitoring the efficacy of RHC therapy, surveillance of remote metastases, and surgical planning. To standardize the assessment of sarcoma progression, the Response Evaluation Criteria in Solid Tumors (RECIST) were created in 2000 and revised in 2009 (RECIST 1.1) [16,17]. However, these criteria only address tumor size, and do not consider tumor viability. To compensate for the lack of information about tumor viability or vascularity, the RECIST 1.1 were modified, resulting in modified RECIST criteria that define viable tumors by considering uptake of contrast agents during the arterial phase of dynamic CT or MRI [18].

Accurate assessment of the efficacy of STS treatment remains controversial compared to that of other solid tumors due to the heterogeneity and rarity of this malignancy. Thus, in the clinical setting, these versatile criteria may complicate the assessment of acquired images, particularly when evaluating the efficacy of neoadjuvant therapy. In this study, we determined the optimal response criteria to evaluate the efficacy of RHC by comparing preoperative images before and after RHC with pathological evaluation of necrosis in the resected tumor.

## Materials and methods

We administered RHC therapy to patients with high-grade (Fédération Nationale des Centres de Lutte Contre le Cancer, grade 2–3) STS in a clinical trial (UMIN000013056). All of the patients were fully informed about RHC therapy and potential associated adverse events, and provided consent to participate. To minimize adverse events, the following exclusion criteria were used: (1) age, 15–70 years, (2) white blood cell count, >3 x 10^6^/mL, (3) platelet count, >7.5 x 10^7^/mL, (4) hemoglobin, >70 g/L, (5) creatinine clearance, >60 mL/min, (6) normal hepatic function, and (7) ejection fraction, >60%.

From 2004 to 2014, We performed RHC for total 27 patients. We excluded 3 patients who underwent unplanned tumor excision, 3 patients who did not undergo definitive surgery and 1 patient who aborted RHC because of severe side effect of chemotherapy. Finally, total 20 patients were enrolled into this study. The mean follow-up period was 60.2 months (range, 12.8–131.6 months). The mean age was 53.0±10.6 years, and there were 14 males and 6 females. Tumor characteristics were diagnosed by needle biopsy prior to RHC therapy, revealing 6 cases of myxoid liposarcoma, 6 cases of undifferentiated pleomorphic sarcoma (UPS), 4 cases of myxofibrosarcoma, and 4 cases of synovial sarcoma (Table 1, S1 Table).

**Table 1:** Clinicopathologic and treatment characteristics of patients undergoing RHC therapy.

	Characteristics	Number	Percentage
Gender	Male	14	70%
	Female	6	30%
Age at diagnosis, years	53.0±10.6 (mean ± standard deviation)		
Stage, UICC 7^th^ edition	2a	3	15%
	2b	6	30%
	3	10	50%
	4	1	5%
Histology	UPS	5	25%
	Myxoid liposarcoma	6	30%
	Synovial sarcoma	5	25%
	Myxofibrosarcoma	4	20%
Location	Thigh	14	70%
	Knee	2	10%
	Lower leg	1	5%
	Forearm	3	15%
Pathologic dimensions	<5 cm	3	15%
	>5 cm	17	85%
Tumor depth	Superficial	7	35%
	Deep	13	65%

UPS, undifferentiated pleomorphic sarcoma

### Study protocol

**Fig 1:**
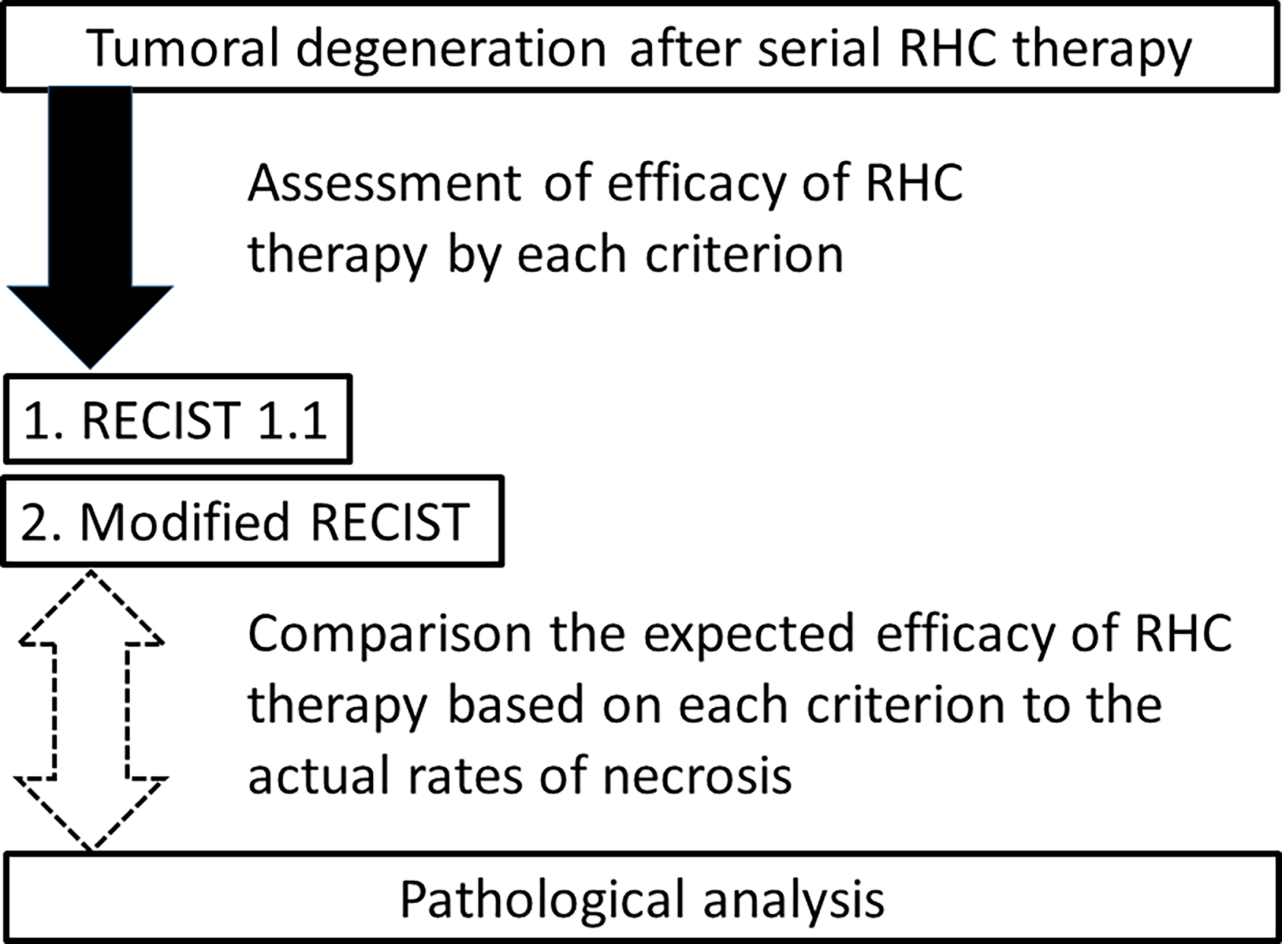
Study protocol.

Before RHC therapy, we performed several assessments to evaluate the length, position, vascularity, and metastasis of tumors using CT or MRI, we repeated similar assessments to evaluate the efficacy of RHC therapy after 3–5 cycles of RHC therapy (post-RHC therapy images). Based on the RECIST 1.1 or modified RECIST, we calculated responses to RHC therapy by comparing pre- and post-RHC therapy images. These criteria are described later in this section. In addition, resected specimens underwent pathological analysis to evaluate response based on tumor necrosis using the four categories [19]. Assessment based on preoperative images and pathological evaluation of necrosis in resected tumor were then compared (Fig 1).

### RHC therapy protocol

**Fig 2:**
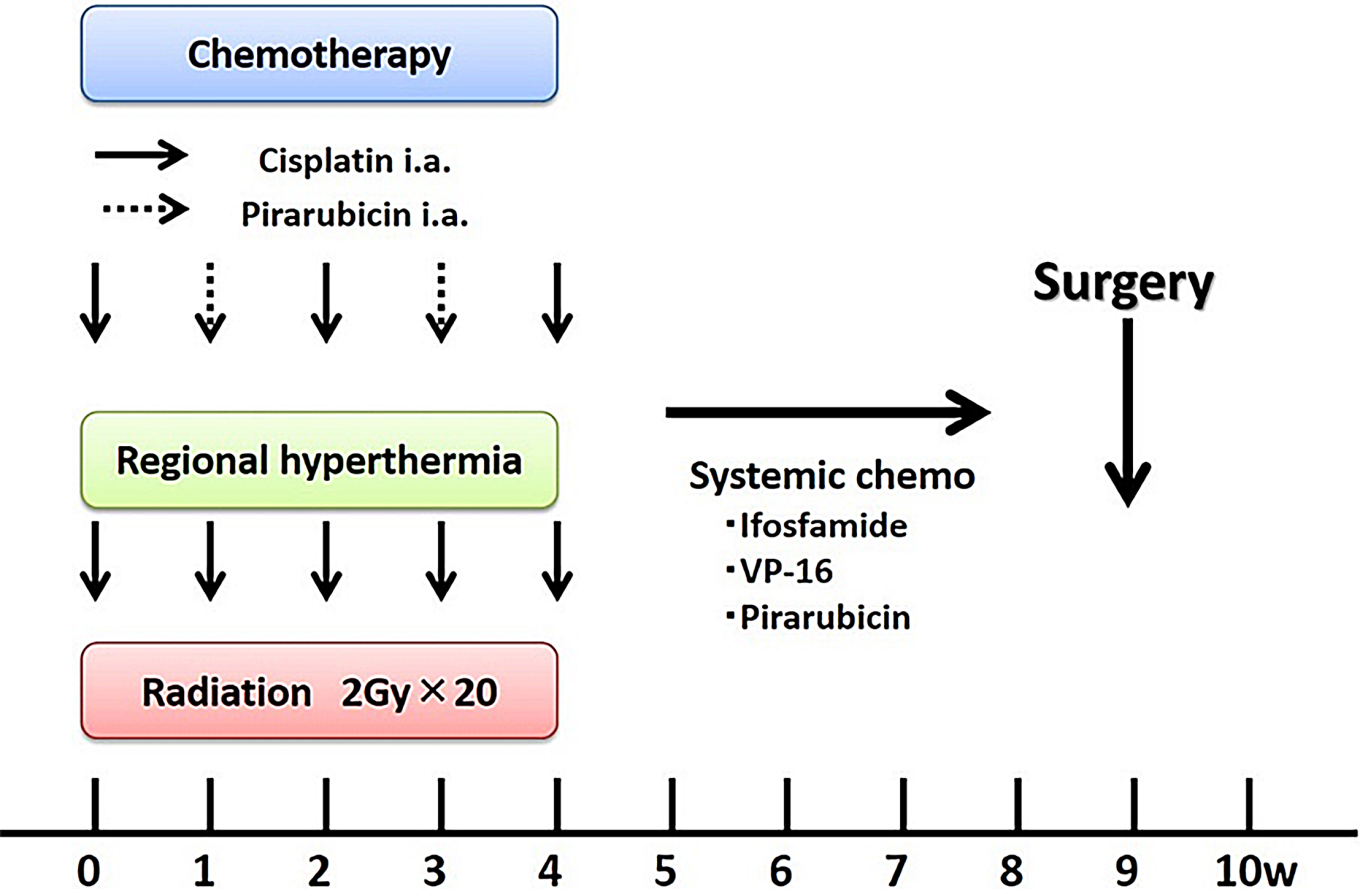
RHC therapy protocol.

Prior to RHC therapy, angiography was performed to assess tumor blood flow, and a catheter was simultaneously inserted with a reservoir placed into the artery feeding the tumor. Placement of a reservoir enabled multiple intra-arterial injections of anticancer drugs during thermotherapy. We alternatively used cisplatin (100 mg/m^2^) on days 1, 15, and 29 and pirarubicin (a doxorubicin derivative; 30 mg/m^2^) on days 8 and 22. Radiotherapy was initiated on Day 1, and 2 Gy per fraction were delivered 20 times (on Days 1–5, 8–12, 15–19, and 22–26) for a total of 40 Gy. Two weeks after RHC therapy (day 43) was completed, chemotherapy (ifosfamide, 2 g/m^2^ [5 days]; etoposide, 100 mg/m^2^ [3 days]; and pirarubicin, 30 mg/m^2^ [2 days]) was administered intravenously. Then, definitive surgery was performed for the primary tumor. More than 2 weeks after RHC therapy was completed, chemotherapy (ifosfamide, 2 g/m^2^ [5 days]; etoposide, 100 mg/m^2^ [3 days]; and pirarubicin, 30 mg/m^2^ [2 days]) was administered intravenously depending on patient status for several cycles. (Fig 2).

For thermotherapy, an 8-MHz radiofrequency capacitive heating system (Thermotron RF-8: Yamamoto VINITA, Osaka, Japan) was used. During thermotherapy, patient temperature was monitored by a sensor inserted into the tumor; the temperature was maintained at 42.5°C for 60 minutes. We defined three categories of hyperthermia quality: complete hyperthermia, completion of ≥4 courses of hyperthermia (42.5°C for 60 minutes); mild hyperthermia, completion of 1–3 courses of hyperthermia; and poor hyperthermia, completion of 0 courses of hyperthermia.

### Criteria used for image evaluation

#### RECIST 1.1

The RECIST 1.1 state that CT is the best currently available and reproducible method to measure lesions selected for response assessment [16, 17]. However, in this study, we also used MRI, because it is particularly appropriate for assessment of soft tissue masses, as the Hounsfield Units of tumor tissue are similar to those of adjacent tissue. The measurement of the greatest longitudinal dimension was calculated by an experienced radiologist, independent of this study. The four response categories included in RECIST 1.1 are complete response (CR), partial response (PR), progressive disease (PD), and stable disease (SD). CR is defined as the disappearance of all target lesions, PR is assigned when the diameter of a target lesion have decreased by >30%, PD indicates an increase of >20% in a target lesion diameter, and SD includes a lesion that does not meet the other criteria.

#### Modified RECIST

Target lesion response was also be evaluated by measuring the percentage change in the length of the viable portion (a portion enhanced during the arterial phase), as is done in modified RECIST [18, 20]. The four response categories included in modified RECIST are CR, PR, PD, and SD. CR is defined as the disappearance of any intratumoral arterial enhancement in a target lesion; PR is assigned when a ≥30% decrease in the length of viable target lesion has occurred, using the baseline of a target lesion diameter as a reference; PD indicates an increase of >20% in viable target lesion, using the diameter of viable target lesion recorded since treatment started as a reference; and SD includes a lesion that does not meet the other criteria.

#### Histological response

Histologic response was defined as follows: Grade 1, little or no effect of chemotherapy observed; Grade 2, partial response to chemotherapy with >50% tumor necrosis; Grade 3, >90% tumor necrosis attributable to preoperative chemotherapy, although foci of apparently viable tumors may be seen in some histologic sections; and Grade 4, no apparent viable tumor cells observed in any of the histologic sections [19].

#### Statistical analysis

All analyses of clinical, biological, and radiographical data were performed by a single observer (H.A). Statistical analysis was performed using SPSS version 24 (IBM^®^). To estimate survival, we used the Kaplan-Meier method, and differences between curves were analyzed by log-rank analysis. For analysis of contingency tables, we calculated the Spearman’s rank-order correlation coefficient with evaluation of the correlation. *P*-values <0.05 were considered statistically significant.

#### Ethics approval

This retrospective study has received the approval of the local committee of Nagoya City University Hospital and has been conducted in compliance with the guidelines of the Helsinki Declaration of 1975.

## Results

As for to the oncologic RHC therapy outcomes, the 3-year survival rate was 86.3%, and the 5-year survival rate was 78.4%. All patients with stage 2a-2b are still alive and 1 patient with stage 4 myxoid liposarcoma, who had received metastasectomy and achieved long-term stable disease with using trabectedin, has been alive with disease for 3.9 years. Among patients with stage 3, the 5-year survival rate was 64.3%. Of 20 patients, 14 patients are clinically disease free (CDF), 3 patients are dead of disease (DOD), and 3 patients are alive with disease (AWD). No recurrences occurred during the study period, although 1 patient had a positive margin. While no deaths occurred among patients with tumors <5 cm, no statistical association between tumor size and risk of death was observed (*P* = 0.425).

### Overall efficacy of RHC therapy

Patient characteristics are shown in Table 1. Overall, 18 of 20 patients underwent >4 rounds of RHC therapy; 2 patients abandoned RHC therapy in the third round due to nausea or vertigo. The quality of hyperthermia was assessed as poor in 6 patients (30%), mild in 7 patients (35%), and complete in 7 patients (35%). Furthermore, the quality of hyperthermia varied across tumor types, although patients with myxoid liposarcomas were more likely to complete hyperthermia (Table 2). No apparent strong correlation was observed between the efficacy of hyperthermia and histological response (Spearman’s Rank-Order Correlation, 0.29; Table 3). In addition, no correlation was observed between histology (Table 4), tumor depth, tumor diameter, or completion of neoadjuvant systemic chemotherapy and success of hyperthermia.

**Table 2:** Relationship between quality of hyperthermia and histology.

Histology						
		UPS	Myxoid liposarcoma	Synovial sarcoma	Myxofibrosarcoma	Total
**Quality of hyperthermia**	Poor	2	0	2	2	6
	Mild	1	3	2	1	7
	Complete	2	3	1	1	7
	Total	5	6	5	4	20

UPS, undifferentiated pleomorphic sarcoma

**Table 3:** Relationship between quality of hyperthermia and histological response.

Histological Response						
		Grade 1	Grade 2	Grade 3	Grade 4	Total
**Quality of hyperthermia**	Poor	0	4	1	1	6
	Mild	1	3	2	1	7
	Complete	1	2	3	1	7
	Total	2	9	6	3	20

No strong correlation between the efficacy of hyperthermia and histological response was observed.

**Table 4:** Relationship between histology and histological response.

Histology						
		UPS	Myxoid liposarcoma	Synovial sarcoma	Myxofibrosarcoma	Total
**Histological response**	Grade 1	0	1	1	0	2
	Grade 2	2	0	4	3	9
	Grade 3	1	4	0	1	6
	Grade 4	2	1	0	0	3
	Total	5	6	5	4	20

UPS, undifferentiated pleomorphic sarcoma

No strong correlation between histology and histological response was observed.

### Correlation between histological response and post-treatment imaging criteria in patients treated with RHC therapy

With respect to RECIST 1.1 (Table 5), the efficacy of RHC therapy was assessed as PD in 1 cases (5%), SD in 14 cases (70%), and PR in 5 cases (25%). In contrast, according to modified RECIST (Table 6), which consider tumor viability, efficacy was assessed as SD in 4 cases (20%), PR in 10 cases (50%), and CR in 6 cases (30%). Spearman’s rank-order correlation for evaluation of the correlation between preoperative images and histological assessment was 0.23 (*P* = 0.33) for RECIST and 0.76 (*P* = 0.001) for modified RECIST. Interestingly, modified RECIST did not underestimate histological responses to RHC therapy.

**Table 5:** Correlation between histological response and response according to various preoperative image-based criteria to RHC therapy- Relationship between RECIST 1.1 and histological response.

Histological Response						
		Grade 1	Grade 2	Grade 3	Grade 4	Total
**RECIST 1.1**	PD	0	0	1	0	1
	SD	2	8	2	2	14
	PR	0	1	3	1	5
	CR	0	0	0	0	0
	Total	2	9	6	3	20

PD, progressive disease; SD, stable disease; PR, partial response; CR, complete response

Note that if the response to RHC therapy was considered to be PD or SD based on RECIST 1.1, it may not necessarily reflect a poor response: 33.3% of SD and PD cases were good responders; Spearman's rank-order correlation, 0.23 (*P* = 0.33).

**Table 6:** Correlation between histological response and response according to various preoperative image-based criteria to RHC therapy- Relationship between modified RECIST and histological response.

Histological Response						
		Grade 1	Grade 2	Grade 3	Grade 4	Total
**Modified RECIST**	PD	0	0	0	0	0
	SD	1	3	0	0	4
	PR	1	6	3	0	10
	CR	0	0	3	3	6
	Total	2	9	6	3	20

PD, progressive disease; SD, stable disease; PR, partial response; CR, complete response

The correlation between modified RECIST and histological response was superior to that for RECIST 1.1. Only 1 case was overestimated as PR despite being Grade 1; Spearman’s rank-order correlation, 0.76 (P<0.001).

### Correlation between efficacy of RHC therapy and oncologic outcome

Responses to RHC therapy were pathologically assessed as Grade 1 in 2 cases (10%), Grade 2 in 9 cases (45%), Grade 3 in 6 cases (30%), and Grade 4 in 3 cases (15%). Despite the lack of a statistical difference (*P* = 0.134; S1 Fig), no deaths occurred in the good responder group (Grade 3–4). Based on evaluation of preoperative images, a good response to RHC therapy appeared to result in a good prognosis.

### Case presentation

**Fig 3:**
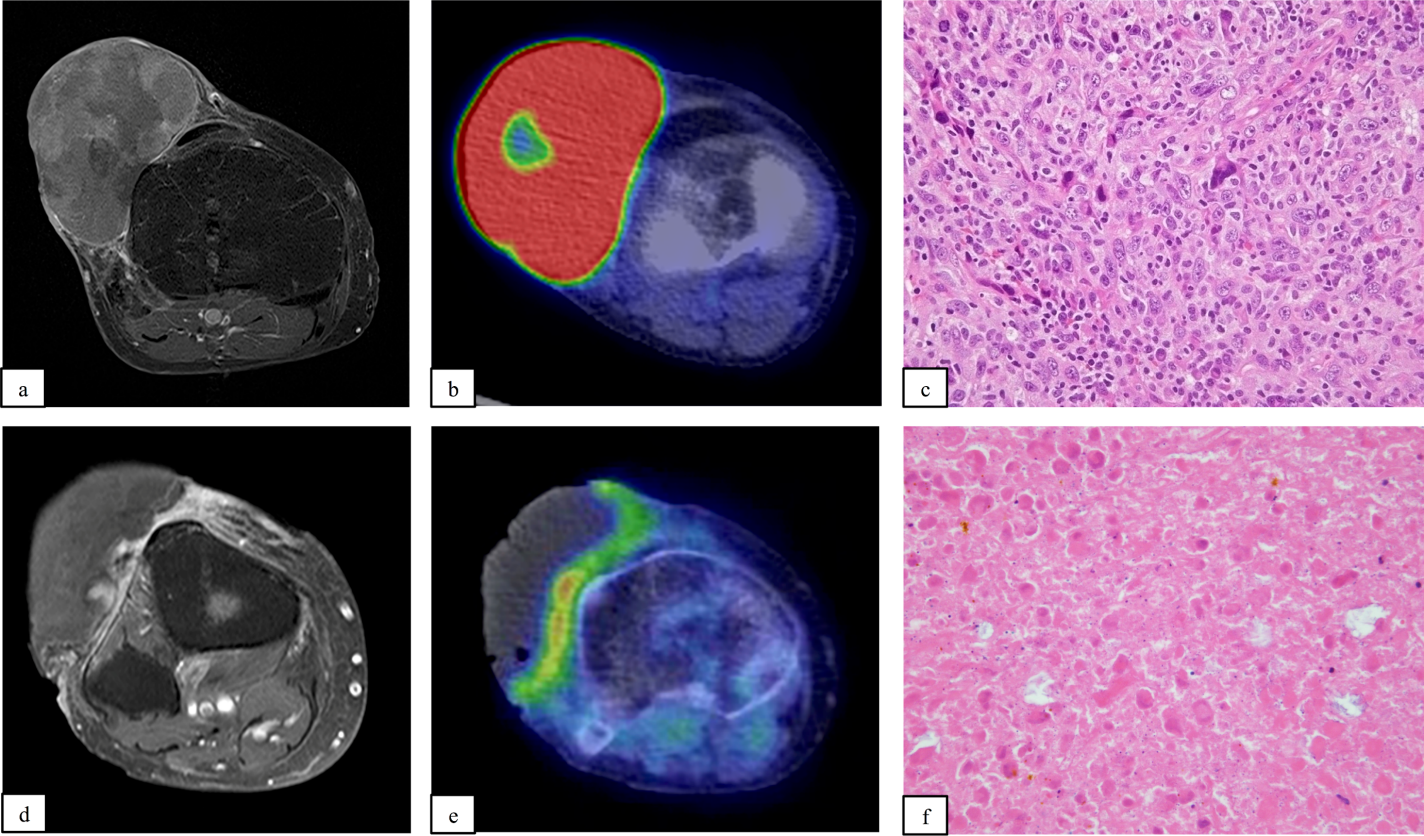
A typical patient treated with RHC therapy (patient no. 10).

**Fig 4:**
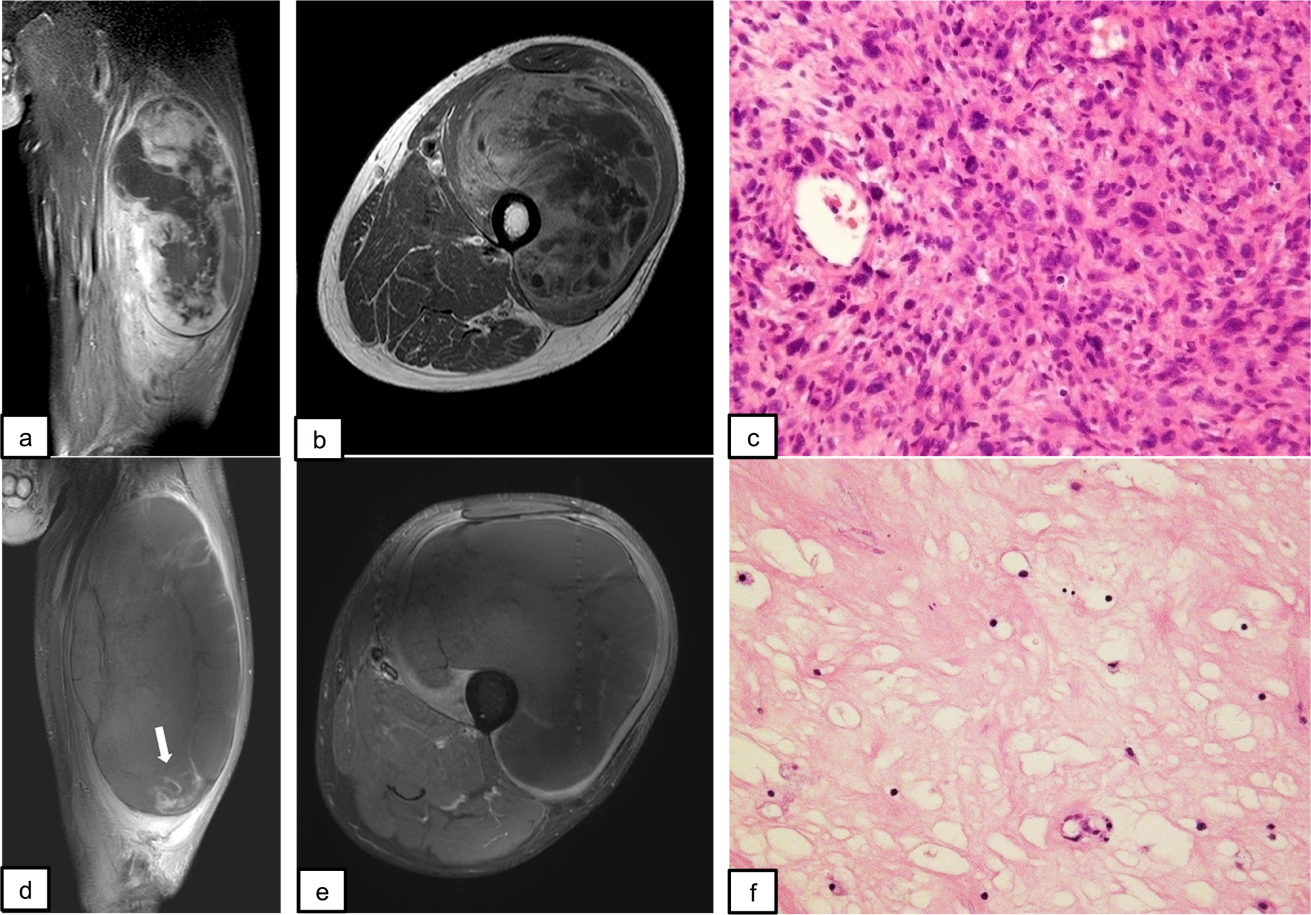
A case in which there was discrepancy between response according to RECIST 1.1 and histological assessment (patient no. 20).

A representative case is shown in Fig 3. Typically, tumor shrinkage was associated with attenuation of tracer uptake, resulting in a decreased contrast agent area. However, RECIST 1.1 sometimes underestimated the efficacy of RHC therapy. Pre- and post-RHC therapy images from a patient with UPS in the right thigh are shown in Fig 4. During the procedure, the tumor had expanded markedly and was assessed as PD based on RECIST 1.1. However, in contrast MRI, the contrast effect had almost disappeared with marginal enhancement and tumor shrinkage.

61-year-old female. The pre-RHC image revealed a soft tissue mass in front of the knee joint that was highly contrasted with gadolinium (a). The standard uptake value (SUV) max was 25.6 (b). Needle biopsy revealed highly malignant UPS. The MIB1 index was 60% (c). After 5 rounds of complete hyperthermia, the lesion had markedly decreased in size. No contrast agent enhancement (d) or tracer uptake (e) was visible after treatment. The efficacy of RHC therapy was considered to be PR by RECIST 1.1 and CR by modified RECIST. Histological analysis showed no viable cells in the lesion; thus, the efficacy of RHC therapy was considered to be Grade 4 (f).

60-year-old male. A giant mass was present in the quadriceps (a-b). The diameter was 110 mm × 88 mm × 178 mm, and the lesion was heterogeneously contrasted with gadolinium. The needle biopsy specimen revealed clusters of mitotically active spindle-shaped, pleomorphic cells (c). The MIB-1 index was 60%. Thus, this lesion was diagnosed as high-grade UPS. After 5 courses of complete hyperthermia, the tumor expanded to 134 mm × 137 mm × 229mm (d-e), with marginal enhancement of the residual tumor (white arrow, d). The efficacy of RHC therapy was considered to be PD by RECIST 1.1 and PR by modified RECIST. Pathologically, >90% of the lesion was degenerated and showed fibrous tissue characteristics. Viable cells were scattered in some parts. Histological evaluation suggested the lesion was Grade 3 (f).

## Discussion

In this study, we analyzed the utility of the response criteria for predicting the efficacy of RHC. Based on histological assessments, 90% of patients responded to RHC (i.e., necrosis in ≥50% of the tumor area was observed), and the modified RECIST were the most accurate criteria for detection of histological changes compared with the other criteria. On the other hand, the RECIST 1.1 criteria sometimes underestimated the histological responses.

Responses to RHC were pathologically assessed as Grade 1 in two cases (10%), Grade 2 in nine cases (45%), Grade 3 in six cases (30%), and Grade 4 in three cases (15%). Malignant cells in surgical specimens from patients with Grade ≥3 tumors were fully degenerated and shrunk rendering the boundaries between the tumor and surrounding tissue more apparent. This can facilitate surgery, resulting in a good local control rate for RHC.

The assessment of necrosis in tumors treated with radiotherapy has been controversial; Willet et al. reported that 21 of 27 patients exhibited necrosis in ≥80% of the tumor in response to 52.5 Gy radiation [21]; on the other hand, Hew et al. observed necrosis in >80% of the tumor in only 35% of patients treated with a once-daily radiotherapy regimen [22]. As for the radiographic response to neoadjuvant radiotherapy, Canter et al. used the RECIST 1.1 and detected SD in 80% and PD in 20% of 25 patients in response to preoperative radiotherapy, given in 2.0-Gy fractions over 25 sessions for a total dose of 50 Gy [23]. This result was similar to that of the current study in that it was difficult to predict actual pathological changes using the RECIST 1.1. To assess the local control rate of preoperative radiotherapy, O’Sullivan et al. performed a relatively large-scale study from 1994 to 1997 [24], which showed that among 94 patients treated with preoperative therapy, the local recurrence rate after surgery was 14% at 1 year and 22% at 2 years. Nevertheless, comparing these results with those of the current study was difficult, as the potential synergistic effect achieved by the three modalities of RHC may have affected the results.

Regarding reports on preoperative chemotherapy, Lucas et al. evaluated the histological response rate pathologically using surgical specimens after a preoperative chemotherapy regimen consisting of four cycles of doxorubicin (60 mg/m^2^) and ifosfamide (2‒3 g/m^2^) [25]. Only 19% of the patients showed an excellent response (>95% necrosis), 10% a moderate response (50‒95% necrosis), and 71% a poor response (<49% necrosis). Regarding the radiographic response, in the Musculoskeletal Tumors Cooperative Study, Ueda et al. reported changes in tumor size based on the Japanese Orthopaedic Association radiographic response evaluation criteria to preoperative chemotherapy, a similar version of RECIST 1.1 for the Japanese. Among 48 patients with malignant STS, 2 (4.1%) achieved a CR, 9 (18.6%) achieved a PR, 32 (66.7%) had SD, and 5 (10.4%) had PD [26]. This result also showed the changes in the tumor size after neoadjuvant chemotherapy were difficult to be evaluated by using RECIST 1.1.

To prevent unnecessary therapy and to minimize the possible adverse events caused by RHC, we carefully monitored changes in the size and viability of tumors during the RHC procedure. Various comorbidities of RHC, including side effects of the hyperthermia such as skin burns and prolonged wound healing, were apparent. In the present study, two patients experienced skin burns, four patients experienced delayed skin healing, and one patient experienced an infection (the skin burn and delayed skin healing cases overlapped). All of these comorbidities were resolved following procedures such as re-suturing and skin flap surgery. In retrospect, two of the patients with Grade 1 disease were not good candidates for RHC. For this reason, we placed greater emphasis on the careful monitoring of responses to RHC.

A limitation of this study is the small number of patients. Due to the rarity of malignant soft tissue tumors, and the cost of installing hyperthermia devices, it would be difficult to perform similar studies in other facilities. However, if the procedure was more standardized or the availability of the apparatus more ubiquitous, we could evaluate the efficacy of RHC in a larger patient population.

Second, we only acquired early-phase contrast MRI. Because it has been suggested that early-phase indicates the tumor vascularity and late-phase the tumor viability [27], the tumor with high vascularity can be overestimated under the current criteria.

Third, selection bias might be an issue in this study, in that 30% of all cases were myxoid liposarcomas, and this subtype exhibits radiosensitivity [28]. Thus, the favorable results might have been due to preoperative RT alone. While the addition of hyperthermia and chemotherapy might be superfluous for local control, their addition might be sufficient to prevent potential distant metastasis. Actually, because of the high rate of response of myxoid/round cell liposarcoma to ifosfamide-based chemotherapy in the metastatic setting, it was hoped that neoadjuvant or adjuvant chemotherapy for high-risk primary liposarcoma would help decrease the frequency of distant metastases and, thus, increase overall survival [29]. As shown in Table 4, the histological response was not specific for myxoid liposarcoma, suggesting that the predominance of myxoid liposarcoma in the study population did not affect the efficacy of RHC in this study.

Finally, in this study, we did not detect a correlation between the quality of hyperthermia and the histological response. This was partially because the histological response was affected by various parameters; e.g. the size of tumor, location or histology, and the efficacy of neoadjuvant therapy was a sum of the trimodal therapy. Actually, Stahl et al. stated the thermal parameters of the specimens were not different between patients with a complete response and those with no response (P = 0.327), when they performed neoadjuvant chemotherapy and hyperthermia [30]. Recently, it was shown that the expression of chemokines and HSP-1 increased the immunological responses after hyperthermia [31]. These are potential reasons for the lack of a correlation between thermal quality and the pathological response.

In conclusion, we performed neoadjuvant RHC for malignant soft tissue tumors and investigated the associations between preoperative imaging and pathological response parameters. We explored the correlations between actual histological responses and the responses dictated by several different imaging criteria. The modified RECIST criteria were more predictive of the efficacy of RHC than were the RECIST 1.1 criteria.

## Supporting information

S1 Table Patients’ details. (DOCX)

S1 Fig Correlation between efficacy of RHC therapy and oncologic outcome. Despite no statistical difference (*P* = 0.134), it is notable that no deaths occurred in the good responder group (Grade 3–4).
(TIF)
