# Supplementary material for: Preoperative evaluation of the efficacy of radio-hyperthermo-chemotherapy for soft tissue sarcoma in a case series
Source: PLoS One. 2018 Apr 16;13(4):e0195289. doi: 10.1371/journal.pone.0195289 (PMC5901917; doi:10.1371/journal.pone.0195289)
Supplement: S1 Table — (DOCX) [file pone.0195289.s001.docx]

| No | Sex | Age  (Years) | Position | Location | Histology | Stage (UICC 7th) | Quality of hyperthermia | Total RHC procedures | Greatest diameter (mm) | | RECIST | Modified RECIST | Pathological Grade | | Prognosis | | Follow-up period  (days) | | Systemic chemotherapy  (preoperative) | Systemic chemotherapy  (postoperative) |
| --- | --- | --- | --- | --- | --- | --- | --- | --- | --- | --- | --- | --- | --- | --- | --- | --- | --- | --- | --- | --- |
|  |  |  |  |  |  |  |  |  | Pre/Post | |  |  |  |  |  |  |  |  |  |  |
| 1 | M | 47.5 | Thigh | Deep | MLS | 2a | Mild | 5 | 44.3 | 30.8 | PR | PR | | Grade 3 | | CDF | | 3949 | + | + |
| 2 | F | 36.7 | Knee | Superficial | MLS | 2b | Mild | 5 | 85.5 | 63.5 | SD | CR | | Grade 3 | | CDF | | 3716 | + | + |
| 3 | M | 65.2 | Thigh | Superficial | MFS | 2b | Poor | 4 | 74.2 | 39.8 | PR | CR | | Grade 3 | | CDF | | 2112 | + | + |
| 4 | M | 39.4 | Forearm | Deep | SS | 3 | Mild | 4 | 95.0 | 93.5 | SD | SD | | Grade 2 | | DOD | | 608 | + | + |
| 5 | M | 39.7 | Thigh | Deep | UPS | 3 | Poor | 5 | 132.7 | 78.5 | PR | CR | | Grade 4 | | CDF | | 2136 | + | + |
| 6 | M | 47.3 | Thigh | Deep | MLS | 4 | Complete | 5 | 131.0 | 157.1 | SD | PR | | Grade 1 | | AWD | | 1429 | + | + |
| 7 | M | 67.2 | Lower leg | Superficial | MFS | 2b | Mild | 4 | 108.9 | 103.6 | SD | PR | | Grade 2 | | CDF | | 981 | + | + |
| 8 | M | 62.2 | Thigh | Deep | UPS | 3 | Mild | 5 | 294.1 | 235.0 | SD | PR | | Grade 2 | | AWD | | 722 | + | + |
| 9 | F | 37.0 | Thigh | Deep | MLS | 3 | Complete | 4 | 108.1 | 109.2 | SD | PR | | Grade 3 | | CDF | | 730 | + | - |
| 10 | F | 61.0 | Knee | Superficial | UPS | 2a | Complete | 5 | 70.4 | 78.6 | SD | CR | | Grade 4 | | CDF | | 390 | + | - |
| 11 | F | 48.0 | Thigh | Deep | SS | 3 | Poor | 3 | 65.7 | 64.0 | SD | SD | | Grade 2 | | CDF | | 3627 | + | - |
| 12 | M | 67.5 | Thigh | Deep | MFS | 3 | Poor | 5 | 112.0 | 104.9 | SD | PR | | Grade 2 | | CDF | | 2836 | - | - |
| 13 | M | 59.3 | Forearm | Deep | SS | 3 | Complete | 5 | 97.0 | 75.0 | SD | PR | | Grade 2 | | DOD | | 1124 | + | + |
| 14 | M | 62.5 | Thigh | Deep | MLS | 3 | Mild | 4 | 100.0 | 99.0 | SD | CR | | Grade 4 | | CDF | | 3400 | + | + |
| 15 | M | 54.9 | Thigh | Deep | UPS | 3 | Poor | 5 | 100.0 | 84 | SD | SD | | Grade 2 | | CDF | | 3200 | + | + |
| 16 | M | 38.9 | Forearm | Superficial | SS | 2b | Mild | 5 | 30.0 | 30.0 | SD | SD | | Grade 1 | | CDF | | 2560 | + | - |
| 17 | F | 45.8 | Thigh | Superficial | MLS | 2a | Complete | 4 | 54.2 | 29.9 | PR | CR | | Grade 3 | | CDF | | 728 | + | - |
| 18 | M | 41.5 | Thigh | Superficial | SS | 3 | Poor | 4 | 118.1 | 137.2 | SD | PR | | Grade 2 | | DOD | | 1388 | + | - |
| 19 | M | 61.6 | Thigh | Deep | MFS | 2b | Complete | 5 | 201.3 | 116.4 | PR | PR | | Grade 2 | | CDF | | 392 | - | - |
| 20 | M | 60.5 | Thigh | Deep | UPS | 2b | Complete | 3 | 178.2 | 229.3 | PD | PR | | Grade 3 | | AWD | | 384 | - | - |

M, male; F, female; MLS, myxoid liposarcoma; MFS, myxofibrosarcoma; UPS, undifferentiated pleomorphic sarcoma; SS, synovial sarcoma; RHC, radio-hyperthermo-chemotherapy; RECIST, Response Evaluation Criteria in Solid Tumors; PD, progressive disease; SD, stable disease; PR, partial response; complete response; DOD, death of disease; AWD, alive with disease; CDF, complete disease free. The parentheses indicate the PERCIST criterion based on PET-CT.

***Supplement***
