# Supplementary figures and images for: Preoperative evaluation of the efficacy of radio-hyperthermo-chemotherapy for soft tissue sarcoma in a case series
Source: PLoS One. 2018 Apr 16;13(4):e0195289. doi: 10.1371/journal.pone.0195289 (PMC5901917; doi:10.1371/journal.pone.0195289)

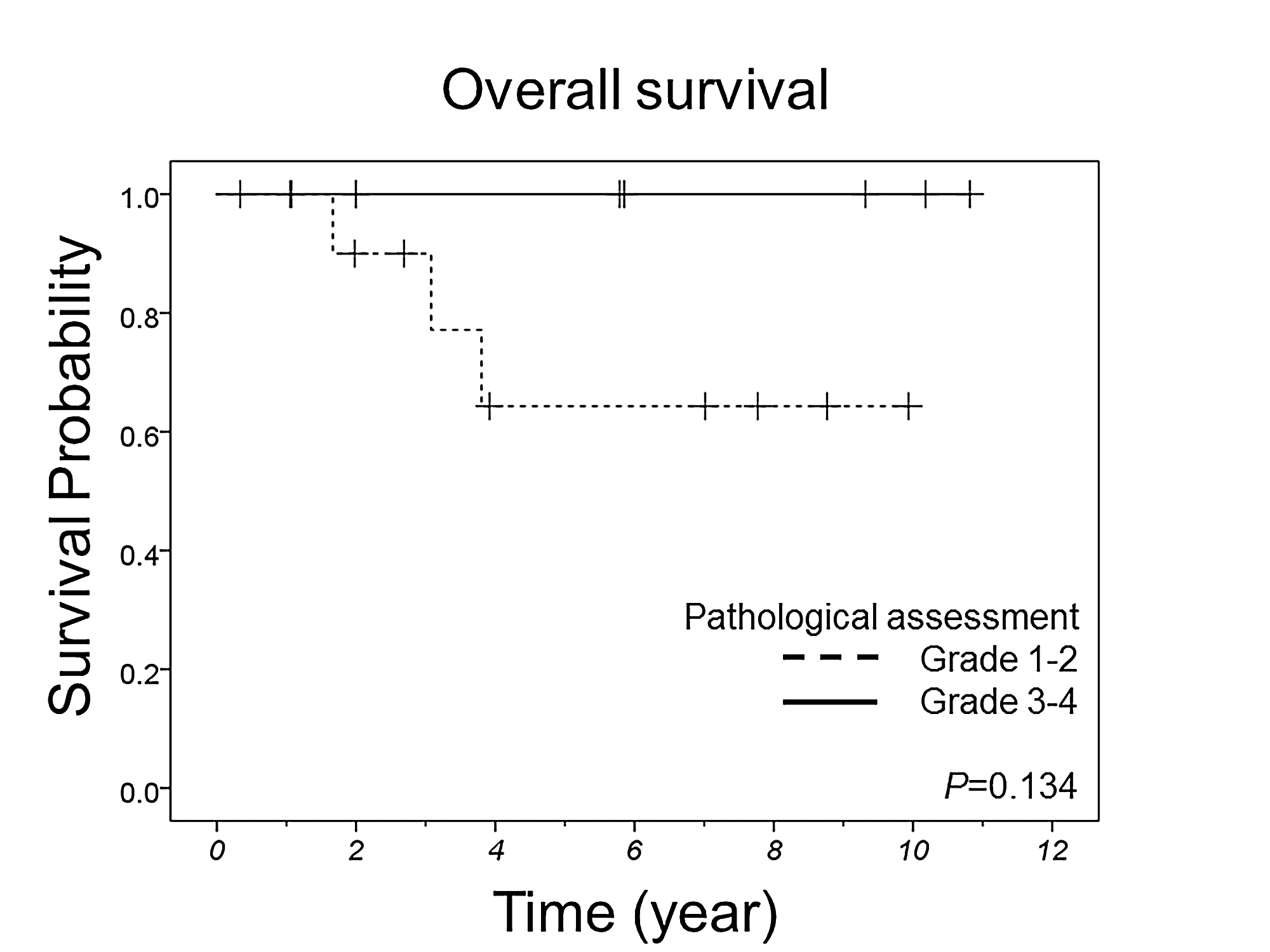

Supplement: S1 Fig — Despite no statistical difference (P = 0.134), it is notable that no deaths occurred in the good responder group (Grade 3–4). (TIF) [file pone.0195289.s002.tif]
